# Strengthening Existing Laboratory-Based Systems vs. Investing in Point-of-Care Assays for Early Infant Diagnosis of HIV: A Model-Based Cost-Effectiveness Analysis

**DOI:** 10.1097/QAI.0000000000002384

**Published:** 2020-06-15

**Authors:** Nicole C. McCann, Jennifer Cohn, Clare Flanagan, Emma Sacks, Sushant Mukherjee, Rochelle P. Walensky, Oluwarantimi Adetunji, Kenneth K. Maeka, Christopher Panella, Addmore Chadambuka, Haurovi Mafaune, Collins Odhiambo, Kenneth A. Freedberg, Andrea L. Ciaranello

**Affiliations:** a Department of Medicine, Medical Practice Evaluation Center, Massachusetts General Hospital, Boston, MA;; b Elizabeth Glaser Pediatric AIDS Foundation, Geneva, Switzerland;; c Division of Infectious Disease, University of Pennsylvania, Philadelphia, PA;; d Elizabeth Glaser Pediatric AIDS Foundation, Washington, DC;; e Division of General Internal Medicine, Massachusetts General Hospital, Boston, MA;; f Division of Infectious Diseases, Massachusetts General Hospital, Boston, MA;; g Harvard University Center for AIDS Research, Boston, MA;; h Early Infant Diagnosis Department, National Microbiology Reference Laboratory, Harare Central Hospital, Harare, Zimbabwe;; i Elizabeth Glaser Pediatric AIDS Foundation, Harare, Zimbabwe; and; j Elizabeth Glaser Pediatric AIDS Foundation, Nairobi, Kenya.

**Keywords:** early infant diagnosis, point-of-care, nucleic acid test, HIV, cost-effectiveness, Zimbabwe, Kenya

## Abstract

**Background:**

To improve early infant HIV diagnosis (EID) programs, options include replacing laboratory-based tests with point-of-care (POC) assays or investing in strengthened systems for sample transport and result return.

**Setting:**

We used the CEPAC-Pediatric model to examine clinical benefits and costs of 3 EID strategies in Zimbabwe for infants 6 weeks of age.

**Methods:**

We examined (1) laboratory-based EID (*LAB)*, (2) strengthened laboratory-based EID (*S-LAB)*, and (3) POC EID (POC). *LAB/S-LAB* and *POC* assays differed in sensitivity (*LAB*/*S-LAB* 100%, *POC* 96.9%) and specificity (*LAB*/*S-LAB* 99.6%, *POC* 99.9%). *LAB/S-LAB/POC* algorithms also differed in: probability of result return (79%/91%/98%), time until result return (61/53/1 days), probability of initiating antiretroviral therapy (ART) after positive result (52%/71%/86%), and total cost/test ($18.10/$30.47/$30.71). We projected life expectancy (LE) and average lifetime per-person cost for all HIV-exposed infants. We calculated incremental cost-effectiveness ratios (ICERs) from discounted (3%/year) LE and costs in $/year-of-life saved (YLS), defining cost effective as an ICER <$580/YLS (reflecting programs providing 2 vs. 1 ART regimens). In sensitivity analyses, we varied differences between *S-LAB* and *POC* in result return probability, result return time, ART initiation probability, and cost.

**Results:**

For infants who acquired HIV, *LAB/S-LAB/POC* led to projected one-year survival of 67.3%/69.9%/75.6% and undiscounted LE of 21.74/22.71/24.49 years. For all HIV-exposed infants, undiscounted LE was 63.35/63.38/63.43 years, at discounted lifetime costs of $200/220/240 per infant*.* In cost-effectiveness analysis, *S-LAB* was an inefficient use of resources; the ICER of *POC* vs. *LAB* was $830/YLS.

**Conclusions:**

Current EID programs will attain greater benefit from investing in POC EID rather than strengthening laboratory-based systems.

## Abstract 2

Supplemental Digital Content is Available in the Text.

## INTRODUCTION

In 2018, 160,000 children became newly infected with HIV worldwide.^1^ Although this is a 40% decrease from the peak of 280,000 new infections in 2010, children living with HIV (CWH) are being left behind in global HIV treatment scale-up; diagnosis and treatment of infant HIV are often missed or delayed.^2^ Because early HIV diagnosis and treatment markedly improve infant survival, the World Health Organization (WHO) recommends early infant diagnosis (EID) testing for all infants exposed to HIV at 6 weeks of age.^3,4^ In 2017, however, only half of infants at risk for HIV received an EID test in the first 2 months of life.^2^ This testing gap is largely due to the need for virological testing in infants, which requires a multistep “cascade of care,” including caregivers bringing infants for testing, sample transport to centralized laboratories, costly nucleic acid laboratory assays, result return to clinics, and return visits for caregivers with infants to receive results.^2^

Improving uptake of each step of the EID cascade is a priority of the WHO and the International AIDS Society, and many countries have implemented various approaches to improve EID outcomes.^5^ One such approach is strengthening laboratory-based EID systems, which may include introducing short message system (SMS) printers and Infant Tracking Systems with text message alerts for mothers, improving sample transport, adding laboratory staff, and increasing laboratory maintenance, to optimize turnaround time and subsequent antiretroviral therapy (ART) initiation.^6–8^ Countries implementing these laboratory-based EID strengthening efforts, including Kenya and Uganda, have seen improvements in result turnaround time and ART initiation after EID testing.^9,10^ Another strategy to improve EID is the use of point-of-care (POC) EID assays, which allow same-day test results and facilitate earlier ART initiation.^11,12^ POC assays are costlier on a per test basis than laboratory-based assays, but are faster and simpler, do not require intensive training or infrastructure, and may be a more efficient means of completing the EID cascade.^13^ POC EID has been shown to be clinically beneficial and cost effective compared with laboratory-based EID,^14^ but the relative clinical impact and cost-effectiveness compared with a strengthened laboratory-based system is unknown.

## METHODS

### Analytic Overview

We used the Cost-Effectiveness of Preventing AIDS Complications (CEPAC)-Pediatric model, a validated Monte Carlo microsimulation of HIV disease,^15^ to project the clinical benefits and costs of 3 testing strategies for infants accessing EID at 6 weeks of age in Zimbabwe: (1) current programs using laboratory-based EID (*LAB*), (2) strengthened laboratory-based EID (*S-LAB*), and (3) replacement of current assays with POC EID (*POC*).

*S-LAB* was defined based on outcomes achieved through an effort in Kenya to improve its EID program, while still using laboratory-based EID tests (see Appendix Figure A, Supplemental Digital Content, http://links.lww.com/QAI/B467). *S-LAB* consisted of an HIV Infant Tracking System with alerts for EID and laboratory staff and mothers, improved sample transport from weekly to daily, additional laboratory staff and training, and increased laboratory maintenance compared with its prestrengthened program.^6–8,16^ *POC* consisted of rapid diagnostic tests offered at all EID sites through the hub and spoke model, in which hub sites, with higher throughput, processed tests on site, and spoke sites, with lower throughput, sent samples to hub sites within 1 hour by all common means of travel. Results from samples processed at hub sites were sent back to spoke sites through SMS printers or phone calls.^12^

We used published and programmatic data to model a cohort of infants born to mothers known to be living with HIV (ie, HIV-exposed infants) who present to 6-week EID testing at prevention of mother-to-child-transmission clinics in Zimbabwe.

For this analysis, model outcomes included short- and long-term survival, life expectancy (LE), and HIV-related costs. Outcomes were evaluated separately for (1) the subset of children who acquired HIV and (2) the total simulated cohort of children who were HIV-exposed. We projected both undiscounted and discounted (3%/year) LE and cost from a health sector perspective. Using the difference in discounted LE and cost between strategies among all children who were HIV-exposed, we calculated the incremental cost-effectiveness ratio (ICER) of each strategy, compared with the next least costly and nondominated alternative. Based on emerging literature, we considered an ICER less than $580 per year of life saved (YLS), the ICER of a program providing 2 vs. 1 lifetime ART regimens to CWH, as cost effective in our base-case analysis.^17–19^ In Zimbabwe, second-line ART is recommended in national HIV pediatric care guidelines.^20^ We used the CEPAC-Pediatric model to determine the ICER of a care strategy that included second-line ART (after failure of first line) compared with a strategy that did not include second-line ART ($580/YLS), as an indicator of health benefits that would be foregone by diverting resources from an existing program to a novel one. Consistent with previous work, we also compared ICER results with the cost-effectiveness threshold of Zimbabwe's 2017 per capita gross domestic product (GDP) ($1600/YLS).^14,21^ In one-way and multiway sensitivity analyses, we varied key model input data and assumptions, including parameters related to assay performance characteristics, test result return, ART initiation, and costs. Base-case parameters were from the Unitaid/EGPAF project, a POC EID testing initiative conducted across 9 African countries from 2015 to 2019, with ranges evaluated in sensitivity analyses from programmatic and published data (see Appendix, p.2, Appendix Table A, Supplemental Digital Content, http://links.lww.com/QAI/B467).

### Model Structure

The CEPAC-Pediatric model is an individual-level, microsimulation computer model of pediatric HIV disease that tracks children from birth through death and projects monthly mortality, LE, and HIV-associated medical costs.^14,15,22–24^ ART availability and maternal CD4 count determine mother-to-child transmission (MTCT) risk, modeled as a one-time risk during the intrauterine and intrapartum periods and a monthly risk during the postpartum period until the end of breastfeeding. CWH experience high mortality before EID testing and subsequent ART initiation. They also face a monthly risk of opportunistic infections (OIs) and risk of mortality from each OI and other HIV-related illnesses. All children are simulated to face monthly risks of non-HIV-related mortality. Planned EID testing can be specified to occur at any age from 0 to 24 months. Upon confirmation of HIV, children experience a probability of linking to HIV care and initiating ART. Once on ART, they face a probability of initial virologic suppression, and subsequently, a monthly risk of treatment failure. Children in care are subject to a monthly risk of becoming lost to follow-up and subsequently a monthly probability of return to care. Additional details are in the Supplemental Appendix, Supplemental Digital Content, http://links.lww.com/QAI/B467 and at https://www.massgeneral.org/medicine/mpec/research/cpac-model**.**

### Modeled Population and Strategies

To reflect the cohort of infants currently presenting to EID programs in Zimbabwe, we simulated infants born to mothers known to be living with HIV. Based on current WHO recommendations, Zimbabwe guidelines, and UNAIDS country reports, we simulated 96% of women receiving ART during pregnancy and breastfeeding (WHO Option B+), with mean breastfeeding duration of 17 months.^4,20,25–27^

We simulated EID testing at 6 weeks for consistency with the Unitaid/EGPAF pilot project and the current structure of most EID programs in sub-Saharan Africa.^12^ For laboratory-based and POC assays, we assigned different diagnostic characteristics (sensitivity and specificity), costs, and uptake of steps in the EID cascade (probability of result return, time to result return, and probability of ART initiation). In the base case, positive laboratory-based or POC results were followed by a confirmatory assay of the same type and opportunity for ART initiation if linked to care. ART was stopped if the confirmatory assay and a third laboratory-based assay (all sent before ART) were negative (see Appendix Figure A, Supplemental Digital Content, http://links.lww.com/QAI/B467). For CWH missed by EID or acquiring HIV after 6 weeks of age, HIV infection could be diagnosed by their later presenting to care for an 18-month clinic visit or at any age with a WHO stage 3 or 4 OI. For these children, PCR testing was used if <18 month of age; HIV antibody testing was used if ≥18 months of age.^4^

### Data Sources

#### Clinical Data

We used recently published peripartum and postpartum MTCT transmission risks.^28^ Mortality rates for children who are HIV-exposed and uninfected were derived from pooled UNAIDS analyses (Table 1).^29–31^ Lacking Zimbabwe-specific data, we used clinical input data calibrated to South African settings for progression of untreated HIV disease.^15,29^ We used International Epidemiologic Database to Evaluate AIDS (IeDEA) East African data and Cape Town AIDS Cohort data to derive CD4 decline, OI, and mortality risk inputs.^15,32,33^ We used data from the P1060 and PENPACT-1 trials to derive probabilities of viral suppression, CD4 increase on suppressive ART, OI, and mortality.^3,34,35^ Clinical input data and calibration are further described elsewhere.^15,35^

**TABLE 1.**
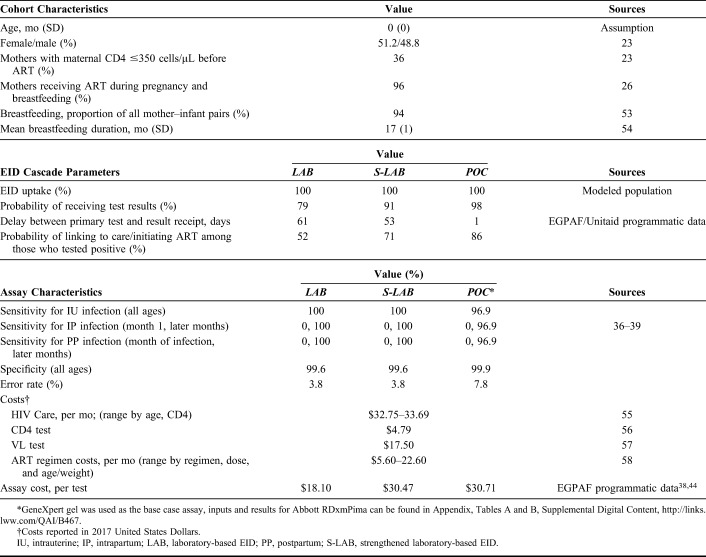
Model Input Parameters

#### Operational Test Characteristics and Care Cascade

We assigned test sensitivity, specificity, and error rates based on EID consortium data and WHO systematic reviews.^36–41^ We derived EID cascade uptake parameters for the *LAB* and *POC* strategies from EGPAF/Unitaid pilot study data in Zimbabwe and derived EID cascade uptake parameters for the *S-LAB* strategy from EGPAF/Unitaid pilot study data in Kenya.^12^ We set this analysis in Zimbabwe because of robust data about the *POC* or *LAB* strategies in that setting. Because no *S-LAB* data were available from Zimbabwe, we used *S-LAB* data from Kenya, assuming that if programs in Zimbabwe strengthened existing laboratory-based systems, they might achieve the result return time, result return probability, and ART initiation probability achieved in Kenya, with application of Zimbabwe-specific costs as described in the *test costs* section below. Based on these data sources, *LAB/S-LAB* and *POC* assays differed in sensitivity (*LAB*/*S-LAB* 100%, *POC* 96.9%) and specificity (*LAB*/*S-LAB* 99.6%, *POC* 99.9%).^37,39–41^ *LAB/S-LAB/POC* algorithms also differed in probability of result return (79%/91%/98%), average time until result return (61/53/1 days),^42,43^ and probability of linking to ART after confirmed positive result (52%/71%/86%). All *POC* input values were weighted averages of results from hub and spoke sites.

#### Test Costs

For the base case, we derived fully loaded costs, inclusive of assay, labor, training, site monitoring, and transport costs, per test for each EID strategy from published costs and a resource utilization analysis in Zimbabwe.^38,44^ The fully loaded cost per test for *LAB* was $18.10.

To estimate the cost per test for *S-LAB* ($30.47), we used programmatic data, collected as part of our resource utilization analysis in Zimbabwe. We first identified the resources required for each component of the strengthening effort in Kenya, then assigned Zimbabwe-specific costs, assuming the same resources would be required in Zimbabwe. The strengthening of the Kenya program included resources for improved specimen transport, SMS printer maintenance, and laboratory staff salary (see Appendix, Supplemental Digital Content, Table D http://links.lww.com/QAI/B467).^6–8^ To reflect a strengthened laboratory-based strategy in Zimbabwe, we adjusted these costs as needed to reach the operational characteristics of strengthened laboratory-based EID in Kenya. This involved calculating the additional cost of increasing sample transport to daily instead of weekly, the cost of adding one EID-specializing laboratory scientist and one EID-specializing junior laboratory officer, and the cost of needed additional training (see Appendix, Table D, Supplemental Digital Content, http://links.lww.com/QAI/B467). The resulting cost of scale-up was $12.71 per test. This cost was then added to the conventional assay cost, excluding overlapping site monitoring, reported by CHAI for a fully loaded per test cost of $30.47.^44^

To derive cost per test for *POC* ($30.71), we used the fully loaded per test cost of the common POC test, GeneXpert Gel, in our base case.^38^ In a scenario analysis, we used the per test cost of the Abbott RDxmPima assay (fully loaded cost = $29.33), which has become available with a reagent rental agreement through which a testing cartridge is purchased at a consolidated cost, inclusive of costs for the platform, maintenance, data, and connectivity, assuming an average of 1300 tests/platform/year (used for on-ART viral load monitoring as well as EID) over 3 years can be attained.^38^ Included in POC per test costs are costs of materials and supplies, training, facility upgrades and repairs, site monitoring and supervision, equipment shipping, labor, and sample transport for the proportion of tests transported between hub and spoke sites (46%). These costs accounted for differences in throughput at hub and spoke sites in Zimbabwe (see Appendix, Table E, Supplemental Digital Content, http://links.lww.com/QAI/B467).

For all assays, we assumed that an error message led the assay to be repeated, with no change in assay return rate or turnaround time. Therefore, assay costs were increased to reflect error rates of 3.8% (LAB), 7.8% (GeneXpert Gel), and 6.7% (Abbott RDxmPima).^38^

### Sensitivity Analyses

In one-way sensitivity analyses, we varied *S-LAB* and *POC* result return probability, result return time, probability of ART initiation, and assay cost to reflect setting-specific differences in pediatric ART services and patient and caregiver behavior (Table 3, see Appendix, Table C, Supplemental Digital Content, http://links.lww.com/QAI/B467). We also examined POC assay sensitivity and specificity over a wide range (Table 3, see Appendix, Table C, Supplemental Digital Content, http://links.lww.com/QAI/B467). In addition, we varied parameters that apply equally to all strategies, including breastfeeding duration, ART efficacy, ART costs, and HIV routine care costs (see Appendix, Table C, Supplemental Digital Content, http://links.lww.com/QAI/B467). In multiway sensitivity analyses, we simultaneously varied parameters related to the degree of laboratory strengthening, including time until result return, probability of result return, probability of ART initiation, and cost. We evaluated values for these parameters between those of *LAB* and *POC* to determine the degree of strengthening needed for *S-LAB* to be cost effective compared with *POC*. Data from other countries in the EGPAF/Unitaid project informed plausible parameter ranges in sensitivity analyses.

### Five-Year Analysis: Clinical Outcomes and Budget Impact

We calculated the number of children detected and linked to care for *LAB*, *S-LAB*, *POC* with GeneXpert Gel, and *POC* with Abbott RDxmPima, and the associated budget impact over 5 years. Modeled 5-year costs included EID costs, cost of treatment, CD4/HVL monitoring, and routine care. We next calculated these costs as a proportion of Zimbabwe's HIV budget,^45^ assuming that the number of infants undergoing EID each year, as well as Zimbabwe's HIV budget, was unchanged each year.

## RESULTS

### Base-Case Results: Clinical Outcomes

**FIGURE 1.**
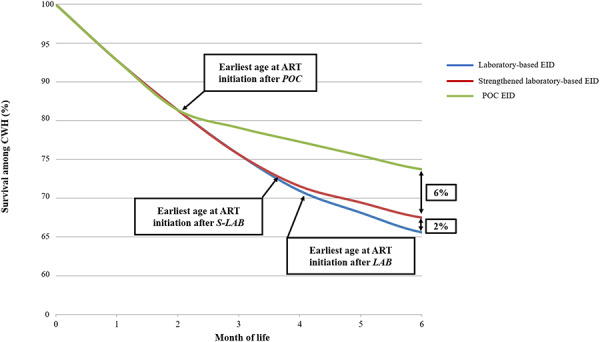
Early survival of CWH survival for CWH through the first 6 months of life, with survival percentage along the vertical axis and time, in months, along the horizontal axis. Survival curves for CWH receiving EID at 6 weeks of age are shown for laboratory-based EID (*LAB*, blue), strengthened laboratory-based EID (*S-LAB*, red), and POC EID (*POC*, green). The point at which infants receive results and initiate ART is marked with arrows for each strategy. The absolute difference in survival between *LAB*, *S-LAB*, and *POC* is shown as a percent at 6 months.

Total projected MTCT for the entire cohort of children exposed to HIV was 3.0%, leaving 97% of the cohort uninfected. Both *S-LAB* and *POC* had large clinical impacts for children who acquired HIV: 1-year survival was 67.3% (*LAB)*, 69.9% (*S-LAB*), and 75.6% (*POC*) and undiscounted LE was 21.74 (*LAB*), 22.71 (*S-LAB*), and 24.49 (*POC*) years. These differences were most notable in the first 6 months of life (Fig. 1). Among the entire cohort of HIV-exposed children, one-year survival was 93.5%, 93.5%, and 93.7%, and undiscounted LE was 63.35, 63.38, and 63.43 years, respectively, for the *LAB*, *S-LAB*, and *POC* strategies (Table 2).

**TABLE 2.**
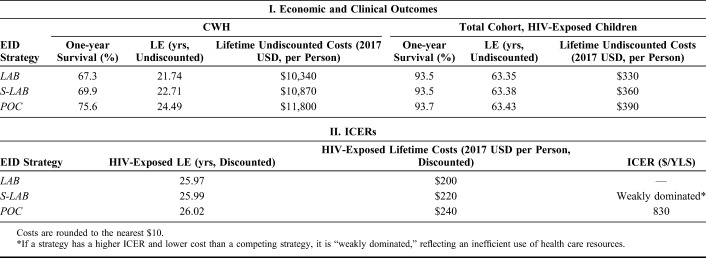
Base-Case Outcomes

### Base-Case Results: Cost and Cost-Effectiveness Outcomes

Among CWH, lifetime-related health care costs were $10,340/infant for *LAB*, $10,870/infant for *S-LAB*, and $11,800/infant for *POC*. The higher cost of the *POC* strategy was primarily attributable to improved initiation on ART and longer LE, with longer durations of costly HIV care and ART. Among the entire cohort of HIV-exposed infants, *LAB* yielded the lowest projected HIV-related health care costs, with a lifetime discounted cost of $200/infant. *S-LAB* yielded a lifetime discounted cost of $220/infant, and *POC* was the costliest strategy, yielding a lifetime discounted cost of $240/infant (Table 2). In cost-effectiveness analysis using discounted outcomes from the entire HIV-exposed cohort, *S-LAB* was a less efficient use of resources than the other strategies (weak dominance^46^), and the ICER of *POC* compared with *LAB* was $830/YLS (50% of Zimbabwe's *per capita* GDP).

### Scenario Analysis: Abbott RDxmPima Reagent Rental

When *POC* was modeled using Abbott RDxmPima, the small decrease in cost and increase in sensitivity led to slightly better, but very similar clinical and economic outcomes than when *POC* was modeled using GeneXpert (see Appendix, Table B, Supplemental Digital Content, http://links.lww.com/QAI/B467), with an ICER for *POC* vs. *LAB* of $790/YLS.

### One-Way Sensitivity Analyses

In one-way sensitivity analyses, *POC* was more efficient than *S-LAB* across a wide range of result return times, result return probabilities, ART initiation probabilities, assay specificities, and costs of *S-LAB* and *POC* (see Appendix Table C, Supplemental Digital Content, http://links.lww.com/QAI/B467). Across the ranges we examined, *POC* was no longer a more efficient use of resources than *S-LAB* when *POC* assay sensitivity was 70% or lower, *POC* result-return probability was 60% or lower, *POC* ART initiation probability was 50% or lower, *POC* costs were $60 or higher, or when *S-LAB* cost was $10 less than *POC* (Table 3; see Appendix Table C, Supplemental Digital Content, http://links.lww.com/QAI/B467). When *POC* was reduced to $18 per test, *POC* was cost effective compared with the second-line ART threshold. *POC* was also more efficient than *S-LAB* across ranges of parameters that apply to all strategies, including breastfeeding duration, ART efficacy, ART costs, and HIV routine care costs.

**TABLE 3.**
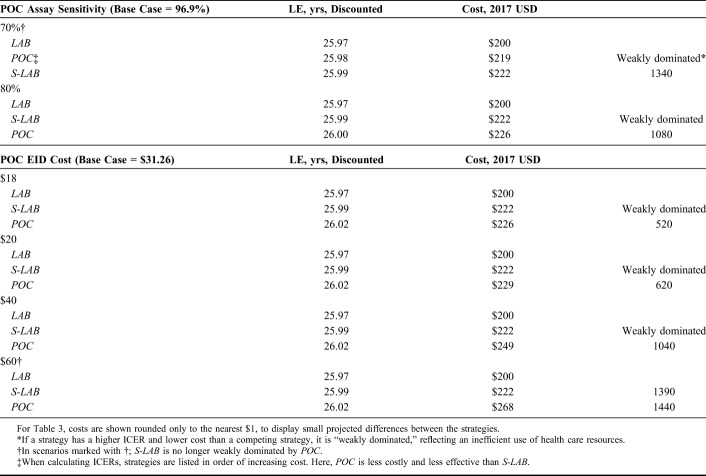
One-Way Sensitivity Analysis: POC Assay Characteristics

### Multiway Sensitivity Analyses

**FIGURE 2.**
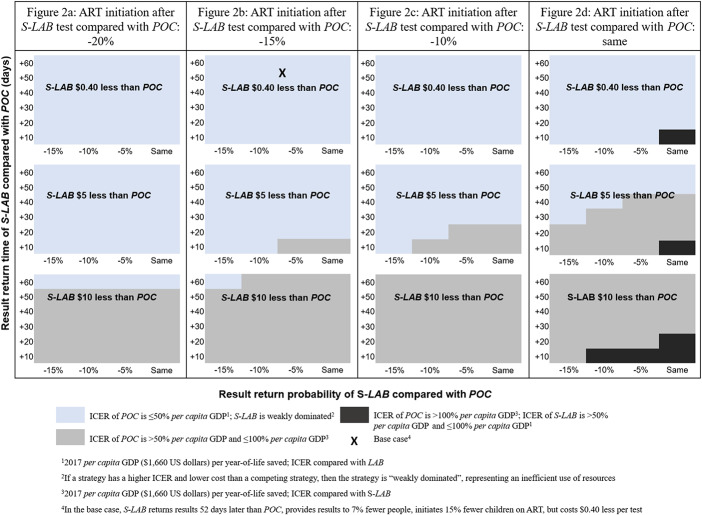
Cost-effectiveness of *POC* as a function of *S-LAB* per test cost, result return time, result return probability, and ART initiation probability. Cost effectiveness of *POC* at varied cost, result return time, result return probability, and ART initiation probability of *S-LAB*. Varied *S-LAB* per test cost is shown in each colored box, from $0.40 less than *POC* to $10 less than *POC*. Along the horizontal axis of each box, result return probability of *S-LAB* is shown, from 15% less than to the same as *POC*. Along the vertical axis of each box, result return time of *S-LAB* is shown, from 60 days longer to 10 days longer than *POC*. Each figure panel shows probability of ART initiation of *S-LAB*, from 20% (A) less than *POC* to the same as *POC* (D). For each combination of parameters, the cost effectiveness of *POC* is shown. Blue: The ICER of *POC* is ≤50% of the per capita GDP; *S-LAB* is weakly dominated. Grey: The ICER of *POC* is >50% of the per capita GDP and ≤100% of the per capita GDP. Black: The ICER of *POC* is >100% of the per capita GDP and *S-LAB* is ≥50% the per capita GDP and ≤100% the per capita GDP. At base-case costs, the ICER of *POC* remained ≤50% of the per capita GDP and the preferred strategy over *S-LAB* unless *S-LAB* result return and ART initiation probabilities were the same as *POC* and result return time was reduced to 10 days. At lower *S-LAB* costs, *POC* was the preferred strategy over *S-LAB* unless *S-LAB* result return time, result return probability, and ART initiation probability were close to those of *POC*, representing marked improvements compared with base-case values. *S-LAB*, strengthened laboratory-base early infant HIV diagnosis.

*POC* remained more efficient than *S-LAB* over a range of *S-LAB* result return times, result return probabilities, ART initiation probabilities, and costs varied between those of *LAB* and *POC*. At the base-case cost, *POC* was always a more efficient use of resources than *S-LAB*, except when result return and ART initiation probabilities were the same as *POC* and result return time was 10 days or less (compared with 53 days in the base case; Fig. 2). Otherwise, for *POC* to no longer be more efficient than *S-LAB*, the per test cost would have to be $5 or $10 dollars less than *POC* while remaining parameters (result return time, result return probability, and ART initiation) approach those of *POC.*

### Five-Year Analysis: Clinical Outcomes and Budget Impact

*LAB* linked 1680 CWH to care and cost 15.7 million over 5 years, using 0.93% of Zimbabwe's entire HIV prevention and treatment budget. *S-LAB* linked 2740 CWH to care and cost 21.6 million over 5 years, using 1.28% of Zimbabwe's HIV budget. *POC* with GeneXpert Gel linked 4480 CWH to care and cost 23.1 million over 5 years, using 1.37% of Zimbabwe's HIV budget. *POC* with Abbott RDxmPima linked 4280 CWH to care and cost 22.7 million over 5 years, using 1.35% of Zimbabwe's HIV budget.

## DISCUSSION

In our model-based analysis examining the most efficient ways to scale up EID in Zimbabwe, we had 4 key findings. First, *POC* reduced early mortality, increased LE, and was a more efficient use of resources than *S-LAB.* Among CWH, *POC* improved clinical outcomes compared with *S-LAB*, increasing 1-year survival by 5.7% and overall LE by 1.8 years; these are substantial improvements at the population level.^47^ *POC* was $40 more costly than *LAB* and $20 more costly than *S-LAB* over the lifetime of each HIV-exposed infant, with most difference in cost attributable to an increase in children taking lifetime ART.

Regardless of the threshold used to assess cost effectiveness, *POC* is a more efficient use of resources than *S-LAB*. If a program is planning to invest in EID, implementing *POC* would be of better value than strengthening existing laboratory-based EID systems. Whether implementing *POC* (rather than continuing *LAB*) is a cost-effective intervention is highly sensitive to the cost-effectiveness threshold. The ICER of *POC* vs*. LAB* ($830/YLS) was above the cost-effectiveness threshold of 2 vs. 1 lifetime ART regimen ($580/YLS), suggesting that more health gains could be obtained from improving access to second-line ART rather than strengthening EID at all. Nonetheless, *POC* may be considered borderline cost effective compared with other HIV-related interventions in Zimbabwe, with an ICER 50% of the annual per capita GDP of $1,600, and also below the ICER of EID programs compared with no EID programs ($1050/YLS).^14^ The affordability of implementing *POC* for all children undergoing EID is a distinct consideration from cost effectiveness. *POC* was projected to link 56% more children to care than *S-LAB*, and to link 155% more children to care than *LAB*, requiring an increase in spending compared with *LAB* equal to only 0.44% of Zimbabwe's total HIV budget.

Second, we found that *POC* is a more efficient use of resources than *S-LAB* across varying levels of laboratory-based EID strengthening. Our base-case results reflect the degree of strengthening achieved in Kenya, assuming the likely costs to conduct the same strengthening activities in Zimbabwe. Because the costs and outcomes of such a program—if it were instead implemented in Zimbabwe—are uncertain, we further examined wide variations in the costs and outcomes of an *S-LAB* program in Zimbabwe. We found that strengthening of a laboratory-based program would need to either achieve almost identical result return probabilities and extremely rapid result return times, or be significantly less costly than *POC* to be an economically valuable alternative to *POC* (Fig. 2). Given that Zimbabwe and Kenya are similarly resourced countries,^21^ it is unlikely that programs in Zimbabwe could implement a strengthened laboratory-based EID program with lower costs, far shorter result return times, and similar or higher ART initiation probabilities than those achieved in Kenya.

Third, if EID programs do not have access to POC EID, and wish to prioritize the most efficient aspects of strengthening laboratory systems, it will be important to understand where bottlenecks occur. Strengthened laboratory-based EID systems have generated result return times as rapid as 7 days in South Africa and Thailand^48,49^; investments might therefore be better allocated to specific aspects of laboratory EID strengthening. To identify bottlenecks, more data are needed about costs and outcomes before and after strengthening in the same laboratory setting.

Fourth, although POC EID assays are costlier than laboratory-based assays, our results are consistent with our previous work and previous reports, demonstrating that the faster result return time, higher result return probability, and higher ART initiation probability associated with POC assays offset plausible differences in cost.^14,50^ Our results show that only at a cost of $60 or greater would *POC* no longer be a more efficient use of resources than *S-LAB*. A total cost of ownership of $60 per POC test has been reported when throughput is low (0.5 tests per day). This is rare in Zimbabwe because POC machines are only placed at high throughput hub sites, with average utilization rates of 1.5 tests per day. When fully loaded cost was reduced to $18 per test, the ICER of *POC* decreased to $520, below the $580 cost-effectiveness threshold. Although implementing *POC* may currently be cost prohibitive in certain settings, reduced pricing, potentially achieved through price negotiation, could render this a more accessible option, even for low throughput settings.

There are several limitations to this analysis. First, clinical care, treatment availability, and HIV-associated costs are likely to change over infants' lifetimes, rendering long-term model-based projections uncertain. We addressed this by calibrating our model to published survival and OI outcomes.^15^ We then varied HIV-related costs, including ART and routine care costs, to account for potential changes over time. Except where noted, plausible changes in these parameters did not change policy conclusions. Second, we modeled a population of infants undergoing EID, that is, 100% EID uptake among HIV-exposed infants for all strategies. This approach excludes the potential benefit of novel programs in improving access to EID testing for infants not currently undergoing testing, as well as the potential role of *POC* or *S-LAB* in testing after 6 weeks of age. In addition, it excludes infants born to women with incident or undiagnosed HIV during breastfeeding. This is likely conservative with regard to the value of *POC*, which may be more likely than *S-LAB* to expand EID access.^51,52^ Finally, some countries are shifting to birth testing for EID, and we did not evaluate the clinical or economic impact of using POC EID at birth. Although recent studies have found POC EID a viable option for birth testing,^49,50^ more research is needed on the clinical and economic outcomes of this strategy compared with laboratory-based EID.

In conclusion, this analysis demonstrated that incorporating POC assays into EID programs at 6 weeks of age in Zimbabwe would reduce early mortality, increase LE, and be a more efficient use of resources than strengthening existing laboratory-based EID programs. Results were robust across a wide range of sensitivity analyses, indicating that they may be generalizable to other sub-Saharan African settings. As POC EID technologies are scaled up, improved access to these assays, strategies to increase linkage to care (such as messaging systems similar to those used in strengthened laboratory scenarios), and lower test costs may further increase the clinical and economic benefit of POC EID. Where POC assays are available, investments in the introduction of these assays will be of better value for EID programs than investing in strengthening existing laboratory-based EID systems.

## Supplementary Material

SUPPLEMENTARY MATERIAL
