# Supplementary material for: Strengthening Existing Laboratory-Based Systems vs. Investing in Point-of-Care Assays for Early Infant Diagnosis of HIV: A Model-Based Cost-Effectiveness Analysis
Source: J Acquir Immune Defic Syndr. 2020 Jun 15;84(1):S12–21. doi: 10.1097/QAI.0000000000002384 (PMC7302325; doi:10.1097/QAI.0000000000002384)
Supplement: SUPPLEMENTARY MATERIAL [file qai-84-s12-s001.docx]

**Early infant diagnosis of HIV:
Strengthening existing laboratory-based systems versus investing in point-of-care assays for early infant diagnosis of HIV: a model-based cost-effectiveness analysis**

Supplementary Appendix

Nicole C. McCann

*Et al.*

**INTRODUCTION**

This appendix is included to provide methodologic details to supplement the methods description in the manuscript text, as well as additional model output and results. For reader convenience, this appendix summarizes information about the CEPAC model structure that has also been described in the technical appendices of prior publications.^1–3^

**EGPAF/UNITAID Collaboration**

This analysis was conducted in collaboration with the Elizabeth Glaser Pediatric AIDS Foundation (EGPAF) and UNITAID, who conducted a point-of-care early infant diagnosis testing initiative from August 2015-July 2019 to expand access of POC EID testing platforms across nine African countries: Cameroon, Cote d’Ivoire, Kenya, Lesotho, Mozambique, Rwanda, Swaziland, Zambia, and Zimbabwe.^4–6^ The project aimed to increase access to EID testing coverage, increase the number of children living with HIV whose status is known, and decrease mortality through timely diagnosis and treatment initiation. The preparation and pilot phases of this project involved identification of gaps within the conventional laboratory network and potential POC EID sites, as well as the gradual implementation of POC EID testing devices as directed by each ministry of health and national technical working groups.^6,7^ We used a validated computer model of pediatric disease to examine the clinical benefits and cost-effectiveness of integrating POC EID platforms into Zimbabwe’s EID network compared to strengthening the existing network, and to understand the potential impact of the POC EID project compared to other possible EID interventions. We anticipated findings could inform future monitoring, evaluation, and implementation efforts. Zimbabwe was chosen as the setting for this model-based analysis for several reasons, including availability of data for use in the model, and available micro-costing resource utilization analysis of the implementation of POC EID at Zimbabwean sites. Additionally, data from Kenya were integrated into this analysis because of available data on its strengthened EID system. Because we aimed to compare a pre-strengthened laboratory-based EID system, a strengthened laboratory-based EID system, and a POC EID system, we required data from all three scenarios. We had detailed data on pre-strengthened laboratory-based, POC EID, and micro-costing in Zimbabwe. We had detailed data from Kenya’s strengthened laboratory-based EID system. We used these Kenya data because no similar data were available from Zimbabwe; we assumed that if programs in Zimbabwe strengthened existing central laboratory systems, they may achieve the result return time, result return probability, and ART initiation probability achieved in Kenya, with application of Zimbabwe-specific costs as described in the methods section (Manuscript, p10; p11).

**METHODS**

**Model Structure and Validation**

In this analysis, we used the Cost-Effectiveness of Preventing AIDS Complications (CEPAC)-Pediatric model to evaluate the clinical benefits, costs, and cost-effectiveness of strengthening existing central laboratory-based EID systems compared to implementing POC EID.^1–3,8,9^ We have previously reported on the structure of the CEPAC-Pediatric natural history model, reflecting HIV disease progression in the absence of ART.^8^ In later manuscripts and corresponding appendices, we provided additional detail about HIV treatment and early infant diagnosis in the CEPAC-Pediatric model.^1–3,10^ The CEPAC model has previously been calibrated to fit observed survival and opportunistic infection (OI) data for children and adults living with HIV, both on and off ART treatment.^8,11,12^ These calibration analyses matched CEPAC output to empiric data in the following domains: OI and survival data for children off ART; OI and survival data for adults off and on ART.^8,11,13–17^ Full details of model structure, data sources, and procedures for initiating new collaborative projects are also available on the CEPAC website, at http://www.massgeneral.org/mpec. Here, we highlight the description of key model features from these prior publications and provide additional detail about EID testing in the CEPAC-Pediatric model.^1–3^

*Maternal cohort characteristics and mother-to-child HIV transmission (MTCT)*

The CEPAC-Pediatric model simulates a cohort of infants from birth through death. At the start of each simulation, each infant is assigned a set of maternal characteristics, including maternal HIV status (chronically infected before pregnancy, acutely infected during pregnancy, or not infected). Among chronically or acutely infected mothers, we specify the probability of HIV testing and availability of ART during pregnancy and breastfeeding. Among mothers not infected during pregnancy, we model probability of acute infection during breastfeeding. For this analysis, we included only infants born to mothers with chronic HIV infection whose status became known during pregnancy. Maternal HIV disease status (chronically or acutely infected) and use of ART determine the risk of HIV transmission during three time periods: intrauterine (one-time risk), intrapartum (one-time risk), and postpartum (monthly risk until weaning).

*Untreated pediatric HIV infection*

At the time of infant HIV infection, infants draw from distributions of HIV RNA and CD4 levels; the model uses CD4 percentage (CD4%) for children <5 years old and absolute CD4 count thereafter. Current age and CD4%/count in each month determine the risks of disease progression, including development of acute OIs and death. Without effective ART, CD4%/count declines monthly. The model tracks true CD4%/count and HIV RNA level, although clinical decisions are made based on observed information, such as symptomatic illness or observed CD4%/count or RNA levels (measured according to user-specified laboratory monitoring strategies). In each month, children can remain in care or be lost to follow-up; if they are lost to follow-up, they are assumed to stop ART, and to return to care if a severe OI occurs.

*Treated pediatric HIV infection*

The model includes criteria by which children can initiate first-line ART, including age, observed CD4%/count, and/or development of OIs; in the base case of this analysis, all children are treated after diagnosis and linkage to care, regardless of these characteristics. For each ART regimen, we specify an “efficacy,” defined as the probability of suppressing HIV RNA to <50 copies/mL (c/mL), and the time point by which this occurs (usually 24 or 48 weeks). Each regimen also confers monthly medication costs, as well as gains in CD4%/count for children with suppressed HIV RNA. Children who initially suppress HIV RNA at 24 or 48 weeks face a monthly risk of virologic failure thereafter (“late failure”). Following virologic failure, HIV RNA slowly rises to a “set point” that is determined as a function of HIV RNA level at birth. After virologic failure, there is a user-specified (base case: 12-month) delay until CD4%/count begins to decline at pre-ART rates, leading to increased monthly risks of OIs and death, until the next effective available ART regimen is initiated. For children who fail each modeled line of ART, we assign clinical criteria (number and type of OIs), immunologic criteria (decline in CD4%/count), or virologic criteria (increase in HIV RNA), by which this failure can be detected, as well as the type and frequency of monitoring and confirmatory testing. After observed failure, patients can be switched to the next available line of therapy. We also incorporate a reduction in mortality and OI risks for children on ART, independent of CD4 level and HIV RNA suppression, as observed in adults; this parameter was used for model calibration.^10,18^

Detection and confirmation of first-line ART failure was only possible after more than 24 weeks on ART and was modeled using the criteria below:

- Virologic failure: Observed RNA >1,000 copies/mL, confirmed by a second RNA test at least 1 month after the first.
- Immunologic failure: Observed CD4% <10% (for children <5 years old) or CD4 count <100/μL (for children ≥5 years old), confirmed by a second CD4%/CD4 count test at least 1 month after the first.
- Clinical failure: Observed new or recurring WHO Stage 3 or 4 OI or TB event, confirmed with a CD4%/CD4 count test at least 1 month after the clinical event.

When immunologic or clinical failure was observed, the ART regimen was not switched until failure was confirmed with a subsequent virologic test.

*Model outcomes*

For each simulated infant, the model tracks clinical events, changes in CD4%/count, and the amount of time spent in each health state. After an individual patient has died, the next infant enters the model. Large cohort sizes (30 million), are simulated in order to generate stable model outcomes. Once the entire cohort has been simulated, summary statistics are tallied, including number and type of clinical events, the proportion alive each month, health care costs in each month, and life expectancy. For EID analyses, the model also reports number of children with HIV detected and not detected at key time points, number of each type of EID assay performed, false positive and false negative EID results, and EID assay costs.

*PCR sensitivity*

PCR sensitivity is modeled as a function of time to reflect the threshold of viremia necessary for detection: PCR at 6 weeks detected intrauterine infection, intrapartum infection, and postpartum infections occurring at least 2 weeks prior.

*False positive and false negative test results*

For both laboratory-based and POC assays, we assume conditional independence of the primary and confirmatory tests. We have reported a detailed discussion of assay specificity and confirmatory test strategies in previous work.^2^ For infants initiating care after a false positive result, we assigned costs for 10 years of routine HIV care, ART, laboratory monitoring, and toxicity events (but not costs related to OIs or the clinical impacts of toxicity, such as morbidity or mortality related to ART toxicity or reduced quality of life).

**Data Sources**

*Test and care costs*

Data from published sources and our resource utilization analysis in Zimbabwe were used to derive the total cost per test for each EID strategy.^19^ As described in the main manuscript, data from Zimbabwe were available for POC EID implementation, while data on the central-laboratory strengthening efforts for this analysis came from Kenya. In order to estimate the cost per test for the *S-LAB* strategy ($30.47), we identified the resources required for each component of the successful strengthening effort in Kenya and then assigned Zimbabwe-specific costs, assuming the same resources would be required in Zimbabwe.

Step 1 / Anticipated annual samples: Calculate anticipated number of EID samples (to inform per sample costs) given Zimbabwe’s current EID sites and average daily throughput. (See Table D1)

Step 2 / Calculating per specimen costs to expand coverage of SMS printers: Within the pre-strengthened Zimbabwe scenario, 1,326 sites (85% of 1,560) had access to an SMS printer. Strengthening the system would require increasing this coverage to the remaining 234 sites. Yearly SMS printer costs per site were calculated to be $270.26, considering the cost of the physical printer, paper, shipping, insurance and SMS data costs. Thus, expanding to 100% coverage would require an additional $63,240 in SMS printer-related costs ($270.26 * 234 sites), resulting in a $0.82 per specimen cost ($64,240 / 76,752 specimens). (See Table D2)

Step 3 / Calculating per specimen training costs: In order to ensure that staff are able to effectively use SMS printers and troubleshoot as needed, a training is available for staff who will be interfacing with the machines. Under the strengthened system with expansion to 100% SMS printer coverage, more staff would need to attend this training. The cost of this training was calculated to a total of $20,060 for these additional staff, considering venue costs and meals, transportation, and accommodations for participants. Given the 76,752 annual specimens, the per specimen training cost was calculated to be $0.26 ($20,060 / 76,752 specimens). (See Table D3)

Step 4 / Additional per sample transportation costs: An important component of the *S-LAB* strategy was moving from weekly transportation to daily transportation. Under the *LAB* strategy, the cost per assay (without site monitoring) was $17.76, which included transportation costs under the weekly transport system.^19^ Thus, we needed to calculate what *additional* costs would be incurred by the increased transportation. Transportation costs were broken into three categories: (1) per sample costs that would be expected to increase (2) non-per sample costs that needed to be increased, and (3) costs not affected by the increased transportation.

- For existing per sample costs, these were originally multiplied by 5 (5 weekdays) to account for the increased per sample costs associated with the change from weekly to daily transportation. For example, $737 was spent on fuel with the once weekly transports. With an average of 1,535 samples being transported per week, this leaves a fuel cost of $0.48 ($737 / 1,535 samples) per sample within the weekly model. Shifting to a daily transportation system means the $737 is increased by five-fold, to $3,685. Meanwhile, the number of samples remains constant at 1,535 weekly, rendering a per sample fuel cost of $2.40 ($3,685 / 1,535 samples).
- The non-per sample costs were individually increased, given the perceived needs. For example, under the weekly transportation cost system, the per sample cost for materials was $0.24. Some of these materials, such as the materials to package samples, would remain consistent regardless of transportation increases. These are, therefore, captured within the existing per sample cost of $17.76 and were not increased in this step. Other material costs, such as those related to additional bikes needed in the daily transport model, were increased.
- Finally, costs related to sample and result tracking were not increased. It was assumed that existing sample tracking systems would be able to support the increased transportation frequency without additional investment.

The sum of per sample costs was calculated to be $12.76 and $2.82 under the daily and weekly transportation systems, respectively. Since the $17.76 cost per test under *LAB* already accounted for once weekly transportation costs ($2.82), these transportation costs were subtracted from the per sample cost under the daily transportation system, so as not to double count. This left $9.94 ($12.76-$2.82) as the additional cost incurred by shifting from a weekly to daily transportation model. (See Table D4)

Step 5 / Calculating additional per specimen salary costs: Strengthening the central laboratory-based EID system would require additional staff, including one additional laboratory scientist per EID machine (5 machines in total) and one additional data officer per testing laboratory (3 testing laboratories total). Considering annual salaries, total additional salary costs for laboratory scientist was calculated at $104,400 ($20,880 yearly salary * 5 additional laboratory scientists needed) and for data officers at $25,200 ($8,400 yearly salary * 3 additional data officers needed). In sum, these salary costs result in a per specimen cost of $1.69 (($104,400 + $25,200) / 76,752 specimens). (See Table D5)

Step 6 / Sum calculated per specimen costs: The per specimen costs calculated in steps 2-5 were summed to determine the total costs for strengthening per specimen: $12.71. (See Table D6)

Step 7 / Add total per specimen costs required for strengthening to the original per test costs within the pre-strengthened system: The cost per assay within the pre-strengthened *LAB* scenario was $17.76. Adding the $12.71 per specimen costs required for strengthening rendered a final cost per test of $30.47 within the *S-LAB* scenario. (See Table D7)

Sensitivity analysis

The CEPAC model accounts for first-order uncertainty (between-patient variability) through the microsimulation of large cohorts of patients. Following the guidance of the International Society for Pharmacoeconomics and Outcomes Research, (ISPOR), we examine the impact of second-order uncertainty (uncertainty in data parameters and assumptions) through wide-ranging univariate and multivariate sensitivity analyses on all model input parameters and assumptions. Please see the technical appendix of a previous publication for a detailed explanation of how CEPAC adheres to ISPOR guidance.^2^ The main manuscript contains key one-way and multiway sensitivity analyses, identifying the thresholds for key parameters at which clinical or cost-effectiveness results change.

**RESULTS**

In the main manuscript, we show the results of the base case analysis, as well as sensitivity analyses in which results and policy conclusions differed from the base case. All other sensitivity analyses listed in the Appendix Methods above did not lead to changes in policy conclusions (available upon request), except where noted in the manuscript. Appendix Table B shows results for selected additional sensitivity analyses.

| **Cohort characteristics** | **Value [range examined]** | | | | | | | | **Sources** |
| --- | --- | --- | --- | --- | --- | --- | --- | --- | --- |
| CD4 % at infection (SD) | 45 (10) | | | | | | | | ^8^ |
| Breastfeeding, proportion of all mother-infant pairs (%) | 94 | | | | | | | | ^21^ |
| Exclusively breastfeeding for 1^st^ 6 months (%) | 65 | | | | | | | | ^22^ |
| Mixed breastfeeding for 1^st^ 6 months (%) | 29 | | | | | | | |  |
| Replacement feeding from birth (%) | 6 | | | | | | | |  |
| Mean breastfeeding duration, months (SD) | 17 (1) [6-24] | | | | | | | | ^23,24^ |
| **Mother-to-child transmission parameters** | **Value [range examined]** | | | | | | | | **Sources** |
|  | Maternal chronic HIV | | | | Maternal acute HIV | | | | ^25^ |
| *Intrauterine (IU)/intrapartum (IP) – one-time risk (%)* |  | | | |  | | | |  |
| On ART (60% IU; 40% IP) |  | | | |  | | | |  |
| ART start before pregnancy | 0.26 | | | | 0.26 | | | |  |
| ART start during pregnancy | 1.4 | | | | 1.4 | | | |  |
| Not on ART (80% IU; 20% IP) | 19.7 | | | | 18.0 | | | |  |
| *Postpartum (PP) – monthly risks during breastfeeding (%)* |  | | | |  | | | |  |
| On ART | 0.11 | | | | 0.11 | | | |  |
| Not on ART | 0.89 | | | | 0.89 | | | |  |
| **EID cascade parameters** | **Value [range examined]** | | | | | | | |  |
|  | ***LAB*** | | ***S-LAB*** | | | ***POC*** | | |  |
| EID uptake (%) | 100 | | 100 | | | 100 | | | Modeled population |
| Probability of receiving test results (%) | 79 | | 91 [79-98] | | | 98 [60-100] | | | EGPAF/Unitaid programmatic data |
| Delay between primary test and result receipt, days | 61 | | 53 [10-60] | | | 1.4 [0-6] | | |  |
| Probability of linking to care/ART among those who tested positive (%) | 52 | | 71 [60-85] | | | 86 [40-100] | | |  |
|  | **Value [range examined]** | | | | | | | |  |
| **Abbott RDxmPima- reagent rental assay characteristics** | ***LAB*** | ***S-LAB*** | | ***POC^*^***  **GeneXpert Gel** | | | ***POC^*^***  **Abbott RDxmPima** | |  |
| Sensitivity for IU infection (all ages) | 100 | 100 | | 96.9 [70-100] | | | | 99.1 | ^26^ |
| Sensitivity for IP infection (month 1, later months) | 0, 100 | 0, 100 | | 0, 96.9 [70-100] | | | | 0, 99.1 |  |
| Sensitivity for PP infection (month of infection, later months) | 0, 100 | 0, 100 | | 0, 96.9 [70-100] | | | | 0, 99.1 |  |
| Specificity (all ages) | 99.6 | 99.6 | | 99.9 [90-100] | | | | 99.9 |  |
| Error rate^**^ (%) | 3.8 | 3.8 | | 7.8 | | | | 6.7 | ^27^ |

**Appendix Table A. Model input parameters**

| **Clinical data: untreated, children living with HIV** | **Value (%)** | **Sources** |
| --- | --- | --- |
| CD4% at infection (SD) | 45 (10) | ^28^ |
| Monthly rate of CD4%/CD4 decline, by age |  |  |
| <3 months of age (CD4%, IU/IP infections only) | 4.00 |  |
| 3-59 months of age (CD4%, IU/IP infections only) | 0.05 |  |
| 0-59 months of age (CD4%, PP infections only) | 0.05 |  |
| ≥60 months of age (CD4 cells/μL, any infection type,  range by HIV RNA) | 3-6 cells |  |
| Monthly risk of clinical events (range by CD4%) |  |  |
| <60 months of age |  |  |
| WHO Stage 3 event (except tuberculosis) | 3.3-11.6 | ^15^ |
| WHO Stage 4 event (except tuberculosis) | 1.4-6.4 |  |
| Tuberculosis (any body site) | 0.5-3.8 |  |
| ≥60 months of age |  |  |
| Mild fungal infection | 1.8-3.1 | ^28^ |
| Visceral bacterial infection | 0.0-0.7 |  |
| WHO Stage 3 or 4 visceral disease | 0.0-1.4 |  |
| WHO Stage 3 or 4 mucocutaneous disease | 0.0-2.3 |  |
| Other WHO Stage 3 or 4 disease | 0.0-0.7 |  |
| Other severe disease | 0.2-1.7 |  |
| Other mild disease | 2.4 |  |
| Tuberculosis (any body site) | 0.0-1.7 |  |

**Appendix Table A. Model input parameters (continued)**

| **Clinical data: untreated, children living with HIV** |  |  |
| --- | --- | --- |
| Risk of death within 30 days of clinical event |  |  |
| <60 months of age | 13.5 | ^28^ |
| After WHO Stage 3 or 4 event | 11.1 |  |
| After TB event |  |  |
| ≥60 months of age |  |  |
| Mild fungal infection | 0.5 | ^15^ |
| Visceral bacterial infection | 2.9 |  |
| WHO Stage 3 or 4 visceral disease | 9.2 |  |
| WHO Stage 3 or 4 mucocutaneous disease | 2.4 |  |
| Other WHO Stage 3 or 4 disease | 20.0 |  |
| Other severe disease | 6.7 |  |
| Other mild disease | 0.4 |  |
| TB (any body site) | 1.8 |  |
| Monthly risk of HIV-related death (range by age, CD4%/CD4, and history of prior OI | 0.1-40.8 |  |
| Monthly risk of infant mortality among breastfed, HIV-exposed, uninfected infants |  |  |
| 0-2 months | 1.0 | ^14,29^ |
| 3-5 months | 0.4 |  |
| 6-11 months | 0.3 |  |
| 12-17 months | 0.1 |  |
| 18-23 months | 0.1 |  |
| Monthly risk of non-AIDS related mortality (range by age in yearly intervals, sex) |  |  |
| <12 months of age | 0.38-0.46 | ^30^ |
| 12-60 months of age | 0.02-0.05 |  |
| 5-13 years of age | 0.01-0.02 |  |
| 13-18 years of age | 0.01-0.02 |  |
| ≥18 years of age | 0.01-1.60 |  |

**Appendix Table A. Model input parameters (continued)**

| **Pediatric ART clinical inputs** | **Value (%) [range examined]** | | **Sources** |
| --- | --- | --- | --- |
|  | LPV/r/ABC/3TC  (1^st^-line ART) | EFV/AZT/3TC  (2^nd^-line ART) |  |
| ART efficacy: HIV RNA <50c/mL at 24 weeks on ART |  |  |  |
| Ages 0-59 months | 91 [90-96] | 75 | ^16,17,33^ |
| Ages 60+ months | 82 [90-96] | 82 |  |
| CD4% gain/CD4 gain on suppressive ART (1^st^ 6 months, after 6 months) |  |  |  |
| Ages 0-59 months | 1.9, 0.4 | 2.2, 0.7 | ^16,17,33^ |
| Ages 60+ months | 77.3, 4.0 | 83.2, 4.2 |  |
| Probability of virologic failure after initial suppression |  |  |  |
| Ages 0-59 months | 0.91 | 0.91 | ^16,17,34^ |
| Ages 60+ months | 0.72 | 0.72 |  |
| **ART outcomes** | **Value (%)** | | **Sources** |
| Relative risk reduction for patients on ART |  | |  |
| Risk of opportunistic infection (age 0-13) | 85 | | ^10^ |
| Risk of opportunistic infection (age 13+) | 32 | | ^18^ |
| Mortality risk (age 0-13) | 90 | | ^10^ |
| Mortality risk (age 13+, range by CD4) | 55-96 | | ^18^ |
| Monthly loss to follow-up after ART initiation | 0.2 | | ^35^ |

**Appendix Table A. Model input parameters (continued)**

**Appendix Table A. Model input parameters (continued)**

| **Costs** | **Value (2017 USD) [range examined]** | **Sources** |
| --- | --- | --- |
| HIV care, children (per month; age <2 years) | $33.69 [0.5x – 3x] | ^36,37^ |
| HIV care, children (per month; age >2 years) | $33.69 [0.5x – 3x] |  |
| HIV care, adults (per month; CD4 vhi, hi, mhi) | $32.75 [0.5x – 3x] |  |
| HIV care, adults (per month; CD4 mlo, lo, vlo) | $32.75 [0.5x – 3x] |  |
| CD4 test | $4.79 | ^38^ |
| VL test | $17.50 | ^39^ |
| Antiretroviral regimen costs (per month, range by age/weight) |  |  |
| LPV/r+ABC+3TC (1st-line ART, all children <13y) | $10.55-22.62 [0.5x – 3x] | ^40^ |
| NVP+AZT+3TC (2nd-line ART, children age <3y) | $5.49-10.05 [0.5x – 3x] |  |
| EFV+AZT+3TC (2nd-line ART, children age ≥3y) | $7.43-8.69 [0.5x – 3x] |  |
| LPV/r+AZT+3TC (1st line ART, adults) | $26.60 [0.5x – 3x] |  |
| EFV+TDF+3TC (2nd line ART, adults) | $8.50 [0.5x – 3x] |  |
| Cost of laboratory-based strategy, per test | $18.10 | ^19^ |
| Cost of strengthened laboratory-based strategy, per test | $30.47 | ^EGPAF programmatic data^ |
| Cost of POC* strategy, per test, GeneXpert Gel | $30.71 | ^27^ |
| Cost of POC* strategy, per test, Abbott RDxmPima | $29.33 |  |

Abbreviations: **SD:** standard deviation; **ART:** antiretroviral therapy; **IU:** intrauterine; **IP:** intrapartum; **PP:** postpartum; **EID:** early infant HIV diagnosis; **POC:** point-of-care; **HIV:** human immunodeficiency virus; **RNA:** ribonucleic acid; **WHO:** World Health Organization; **TB:** tuberculosis; **OI:** opportunistic infection; **LPV/r:** lopinavir/ritonavir; **ABC:** abacavir; **3TC:** lamivudine; **EFV:** efavirenz; **NVP:** nevirapine; **AZT:** zidovudine; **USD:** United States dollar; **VL:** viral load.

^*^Two *POC* scenarios were modeled. In the base case, GeneXpert Gel was used, and in a scenario analysis Abbott RDxmPima with rental reagent agreement price was used.

^**^Error in performing a POC test (due to a platform malfunction, human error, etc.) leads to an inconclusive test result and a repeat test, but does not affect result return.

| **II. Incremental cost-effectiveness ratios (ICERs)** | | | |
| --- | --- | --- | --- |
| **EID strategy** | **HIV-exposed life expectancy**  **(years, discounted)** | **HIV-exposed lifetime costs (2017 USD per person, discounted)** | **Incremental cost-effectiveness ratio ($/YLS)** |
| *LAB* | 25.97 | $200 | -- |
| *S-LAB* | 25.99 | $222 | dominated* |
| *POC* | 26.02 | $240 | 790 |

**Appendix Table B. Reagent rental scenario: Clinical and economic outcomes**

*If a strategy has a higher ICER and lower cost than a competing strategy, it is “weakly dominated”, reflecting an inefficient use of healthcare resources

Costs are rounded to the nearest $1 to display small projected differences from the base case

**Appendix Table C. Extended sensitivity analysis results**

| **Base case** |  |  |  |
| --- | --- | --- | --- |
| EID strategy | **Birth cohort life expectancy**  **(years, discounted)** | **Birth cohort lifetime costs**  **(USD per person, discounted)** | **Incremental cost-effectiveness ratio ($/YLS)** |
| *LAB* | 25.97 | $200 | -- |
| *S-LAB* | 25.99 | $222 | dominated^*^ |
| *POC* | 26.02 | $239 | 830 |
| **Strengthened laboratory-based EID variables** |  |  |  |
| EID strategy | **Birth cohort life expectancy**  **(years, discounted)** | **Birth cohort lifetime costs**  **(USD per person, discounted)** | **Incremental cost-effectiveness ratio ($/YLS)** |
| *Strengthened laboratory-based EID test cost $20.71* |  |  |  |
| *LAB* | 25.97 | $200 | -- |
| *S-LAB* | 25.99 | $212 | 740 |
| *POC* | 26.02 | $239 | 880 |
| *Strengthened laboratory-based EID test cost $25.71* |  |  |  |
| *LAB* | 25.97 | $200 | -- |
| *S-LAB* | 25.99 | $217 | dominated |
| *POC* | 26.02 | $239 | 830 |

| EID strategy | **Birth cohort life expectancy**  **(years, discounted)** | **Birth cohort lifetime costs**  **(USD per person, discounted)** | **Incremental cost-effectiveness ratio ($/YLS)** |
| --- | --- | --- | --- |
| *Strengthened laboratory-based EID result return time 5 days* |  |  |  |
| *LAB* | 25.97 | $200 | -- |
| *S-LAB* | 26.00 | $231 | dominated |
| *POC* | 26.02 | $239 | 830 |
| *Strengthened laboratory-based EID result return time 10 days* |  |  |  |
| *LAB* | 25.97 | $200 | -- |
| *S-LAB* | 26.00 | $230 | dominated |
| *POC* | 26.02 | $239 | 830 |
| *Strengthened laboratory-based EID result return time 20 days* |  |  |  |
| *LAB* | 25.97 | $200 | -- |
| *S-LAB* | 26.00 | $228 | dominated |
| *POC* | 26.02 | $239 | 830 |
| *Strengthened laboratory-based EID result return time 30 days* |  |  |  |
| *LAB* | 25.97 | $200 | -- |
| *S-LAB* | 26.00 | $226 | dominated |
| *POC* | 26.02 | $239 | 830 |
| *Strengthened laboratory-based EID result return time 40 days* |  |  |  |
| *LAB* | 25.97 | $200 | -- |
| *S-LAB* | 25.99 | $224 | dominated |
| *POC* | 26.02 | $239 | 830 |
| *Strengthened laboratory-based EID result return time 60 days* |  |  |  |
| *LAB* | 25.97 | $200 | -- |
| *S-LAB* | 25.99 | $221 | dominated |
| *POC* | 26.02 | $239 | 830 |

**Appendix Table C. Extended sensitivity analysis results (continued)**

| EID strategy | **Birth cohort life expectancy**  **(years, discounted)** | **Birth cohort lifetime costs**  **(USD per person, discounted)** | **Incremental cost-effectiveness ratio ($/YLS)** |
| --- | --- | --- | --- |
| *Strengthened laboratory-based EID result return probability 83%* |  |  |  |
| *LAB* | 25.97 | $200 | -- |
| *S-LAB* | 25.99 | $218 | dominated |
| *POC* | 26.02 | $239 | 830 |
| *Strengthened laboratory-based EID result return probability 88%* |  |  |  |
| *LAB* | 25.97 | $200 | -- |
| *S-LAB* | 25.99 | $220 | dominated |
| *POC* | 26.02 | $239 | 830 |
| *Strengthened laboratory-based EID result return probability 98%* |  |  |  |
| *LAB* | 25.97 | $200 | -- |
| *S-LAB* | 25.99 | $224 | dominated |
| *POC* | 26.02 | $239 | 830 |

**Appendix Table C. Extended sensitivity analysis results (continued)**

| EID strategy | **Birth cohort life expectancy**  **(years, discounted)** | **Birth cohort lifetime costs**  **(USD per person, discounted)** | **Incremental cost-effectiveness ratio ($/YLS)** |
| --- | --- | --- | --- |
| *Strengthened laboratory-based EID ART initiation probability 60%* |  |  |  |
| *LAB* | 25.97 | $200 | -- |
| *S-LAB* | 25.98 | $219 | dominated |
| *POC* | 26.02 | $239 | 830 |
| *Strengthened laboratory-based EID ART initiation probability 65%* |  |  |  |
| *LAB* | 25.97 | $200 | -- |
| *S-LAB* | 25.99 | $220 | dominated |
| *POC* | 26.02 | $239 | 830 |
| *Strengthened laboratory-based EID ART initiation probability 70%* |  |  |  |
| *LAB* | 25.97 | $200 | -- |
| *S-LAB* | 25.99 | $222 | dominated |
| *POC* | 26.02 | $239 | 830 |
| *Strengthened laboratory-based EID ART initiation probability 75%* |  |  |  |
| *LAB* | 25.97 | $200 | -- |
| *S-LAB* | 25.99 | $224 | dominated |
| *POC* | 26.02 | $239 | 830 |
| *Strengthened laboratory-based EID ART initiation probability 80%* |  |  |  |
| *LAB* | 25.97 | $200 | -- |
| *S-LAB* | 25.99 | $226 | dominated |
| *POC* | 26.02 | $239 | 830 |
| *Strengthened laboratory-based EID ART initiation probability 85%* |  |  |  |
| *LAB* | 25.97 | $200 | -- |
| *S-LAB* | 26.00 | $228 | dominated |
| *POC* | 26.02 | $239 | 830 |

**Appendix Table C. Extended sensitivity analysis results (continued)**

| **POC EID variables** |  |  |  |
| --- | --- | --- | --- |
| EID strategy | **Birth cohort life expectancy**  **(years, discounted)** | **Birth cohort lifetime costs**  **(USD per person, discounted)** | **Incremental cost-effectiveness ratio ($/YLS)** |
| *POC EID test cost $10* |  |  |  |
| *LAB* | 25.97 | $200 | -- |
| *POC* | 26.02 | $219 | 400 |
| *S-LAB* | 25.99 | $222 | Dominated^**^ |
| *POC EID test cost $20* |  |  |  |
| *LAB* | 25.97 | $200 | -- |
| *S-LAB* | 25.99 | $222 | dominated |
| *POC* | 26.02 | $229 | 620 |
| *POC EID test cost $40* |  |  |  |
| *LAB* | 25.97 | $200 | -- |
| *S-LAB* | 25.99 | $222 | dominated |
| *POC* | 26.02 | $249 | 1,040 |
| *POC EID test cost $50* |  |  |  |
| *LAB* | 25.97 | $200 | -- |
| *S-LAB* | 25.99 | $222 | dominated |
| *POC* | 26.02 | $258 | 1,230 |
| *POC EID test cost $60* |  |  |  |
| *LAB* | 25.97 | $200 | -- |
| *S-LAB* | 25.99 | $222 | 1,340 |
| *POC* | 26.02 | $268 | 1,440 |

**Appendix Table C. Extended sensitivity analysis results (continued)**

| EID strategy | **Birth cohort life expectancy**  **(years, discounted)** | **Birth cohort lifetime costs**  **(USD per person, discounted)** | **Incremental cost-effectiveness ratio ($/YLS)** |
| --- | --- | --- | --- |
| *POC probability of result return 60%* |  |  |  |
| *LAB* | 25.97 | $200 | -- |
| *S-LAB* | 25.99 | $222 | 1,340 |
| *POC* | 25.99 | $223 | Dominated |
| *POC probability of result return 70%* |  |  |  |
| *LAB* | 25.97 | $200 | -- |
| *S-LAB* | 25.99 | $222 | dominated |
| *POC* | 26.00 | $227 | 1,010 |
| *POC probability of result return 80%* |  |  |  |
| *LAB* | 25.97 | $200 | -- |
| *S-LAB* | 25.99 | $222 | dominated |
| *POC* | 26.01 | $232 | 940 |
| *POC probability of result return 90%* |  |  |  |
| *LAB* | 25.97 | $200 | -- |
| *S-LAB* | 25.99 | $222 | dominated |
| *POC* | 26.01 | $236 | 870 |
| *POC probability of result return 100%* |  |  |  |
| *LAB* | 25.97 | $200 | -- |
| *S-LAB* | 25.99 | $222 | dominated |
| *POC* | 26.02 | $240 | 820 |
| *POC time until result return, 6 days* |  |  |  |
| *LAB* | 25.97 | $200 | -- |
| *S-LAB* | 25.99 | $222 | dominated |
| *POC* | 26.02 | $239 | 810 |

**Appendix Table C. Extended sensitivity analysis results (continued)**

| EID strategy | **Birth cohort life expectancy**  **(years, discounted)** | **Birth cohort lifetime costs**  **(USD per person, discounted)** | **Incremental cost-effectiveness ratio ($/YLS)** |
| --- | --- | --- | --- |
| *POC probability of ART initiation 40%* |  |  |  |
| *LAB* | 25.97 | $200 | -- |
| *POC* | 25.98 | $217 | dominated |
| *S-LAB* | 25.99 | $222 | 1,340 |
| *POC probability of ART initiation 50%* |  |  | -- |
| *LAB* | 25.97 | $200 | -- |
| *POC* | 25.99 | $222 | dominated |
| *S-LAB* | 25.99 | $222 | 1,340 |
| *POC probability of ART initiation* 60% |  |  |  |
| *LAB* | 25.97 | $200 | -- |
| *S-LAB* | 25.99 | $222 | dominated |
| *POC* | 26.00 | $228 | 1,090 |
| *POC probability of ART initiation 70*% |  |  |  |
| *LAB* | 25.97 | $200 | -- |
| *S-LAB* | 25.99 | $222 | dominated |
| *POC* | 26.01 | $231 | 930 |
| *POC probability of ART initiation 80*% |  |  |  |
| *LAB* | 25.97 | $200 | -- |
| *S-LAB* | 25.99 | $222 | dominated |
| *POC* | 26.01 | $236 | 860 |

**Appendix Table C. Extended sensitivity analysis results (continued)**

**Appendix Table C. Extended sensitivity analysis results (continued)**

| EID strategy | **Birth cohort life expectancy**  **(years, discounted)** | **Birth cohort lifetime costs**  **(USD per person, discounted)** | **Incremental cost-effectiveness ratio ($/YLS)** |
| --- | --- | --- | --- |
| *POC specificity 90%* |  |  |  |
| *LAB* | 25.97 | $200 | -- |
| *S-LAB* | 25.99 | $222 | dominated |
| *POC* | 26.02 | $242 | 900 |
| *POC specificity 92%* |  |  |  |
| *LAB* | 25.97 | $200 | -- |
| *S-LAB* | 25.99 | $222 | dominated |
| *POC* | 26.02 | $242 | 900 |

| **Variables applied to all strategies** |  |  |  |
| --- | --- | --- | --- |
| EID strategy | **Birth cohort life expectancy**  **(years, discounted)** | **Birth cohort lifetime costs**  **(USD per person, discounted)** | **Incremental cost-effectiveness ratio ($/YLS)** |
| *HIV care costs 0.5x base case* |  |  |  |
| *LAB* | 25.97 | $138 | -- |
| *S-LAB* | 25.99 | $157 | dominated |
| *POC* | 26.02 | $168 | 640 |
| *HIV care costs 2x base case* |  |  |  |
| *LAB* | 25.97 | $321 | -- |
| *S-LAB* | 25.99 | $350 | dominated |
| *POC* | 26.02 | $380 | 1,250 |
| *HIV care costs 3x base case* |  |  |  |
| *LAB* | 25.97 | $444 | -- |
| *S-LAB* | 25.99 | $480 | dominated |
| *POC* | 26.02 | $521 | 1,630 |
| *ART costs 0.5x base case* |  |  |  |
| *LAB* | 25.97 | $176 | -- |
| *S-LAB* | 25.99 | $197 | dominated |
| *POC* | 26.02 | $212 | 760 |
| *ART costs 2x base case* |  |  |  |
| *LAB* | 25.97 | $247 | -- |
| *S-LAB* | 25.99 | $273 | dominated |
| *POC* | 26.02 | $294 | 1,000 |
| *ART costs 3x base case* |  |  |  |
| *LAB* | 25.97 | $309 | -- |
| *S-LAB* | 25.99 | $337 | dominated |
| *POC* | 26.02 | $364 | 1,170 |

**Appendix Table C. Extended sensitivity analysis results (continued)**

**Appendix Table C. Extended sensitivity analysis results (continued)**

| EID strategy | **Birth cohort life expectancy**  **(years, discounted)** | **Birth cohort lifetime costs**  **(USD per person, discounted)** | **Incremental cost-effectiveness ratio ($/YLS)** |
| --- | --- | --- | --- |
| *1^st^ line ART efficacy 90%, <50c/mL at 24 weeks on ART, all ages* |  |  |  |
| *LAB* | 25.97 | $199 | -- |
| *S-LAB* | 25.99 | $221 | dominated |
| *POC* | 26.02 | $239 | 850 |
| *1^st^ line ART efficacy 96%, <50c/mL at 24 weeks on ART, all ages* |  |  |  |
| *LAB* | 25.97 | $202 | -- |
| *S-LAB* | 25.99 | $224 | dominated |
| *POC* | 26.02 | $242 | 840 |
| *Breastfeeding duration 6 months* |  |  |  |
| *LAB* | 26.09 | $134 | -- |
| *S-LAB* | 26.10 | $157 | dominated |
| *POC* | 26.13 | $174 | 840 |
| *Breastfeeding duration 24 months* |  |  |  |
| *LAB* | 25.90 | $237 | -- |
| *S-LAB* | 25.92 | $260 | dominated |
| *POC* | 25.95 | $277 | 850 |

^*^ If a strategy has a higher ICER and lower cost than a competing strategy, it is “weakly dominated”, reflecting an inefficient use of healthcare resources

^**^If a strategy has a higher cost and is less effective than a competing strategy, it is “dominated”

Costs are rounded to the nearest $1 to display small projected differences

When calculating ICERs, each strategy is compared to the next least costly, non-dominated strategy. Therefore, when *S-LAB* is dominated, *POC* is compared to *LAB* the ICER of *POC* stays stable at $830 / YLS.

**Appendix Table D: Calculating the per test cost of strengthened laboratory-based EID**

Step 1 / Anticipated annual samples

| **Table D1** |  |  |
| --- | --- | --- |
| **Anticipated annual specimens** |  |  |
| EID Sites in Zimbabwe | 1,560 |  |
| Average daily throughout (per site per day) | 0.20 |  |
| Working days/year | 246 |  |
| Annual specimens expected | 76,752 | = (1560) * (0.20) * (246) |

## Step 2 / Calculating per specimen costs to expand coverage of SMS printers

| **Table D2** | |
| --- | --- |
| **SMS printer costs per site per year** | |
| SMS printers | $ 240 |
| Thermal printing paper | $ 20 |
| Freight, shipping & insurance | $ 7.80 |
| SMS data costs (annual) | $ 2.46 |
| **SUM** | $ 270.26 |

| **Calculating per specimen SMS printer cost** | | | |
| --- | --- | --- | --- |
| SMS printer costs per year per site | $270.26 |  |  |
| Current coverage | 85% |  |  |
| Current number of sites not covered | 234 | = 1,560 total sites * (15%) |  |
| Cost per site for sites not currently covered | $63,240 | = ($270.26) * (234 sites) |  |
| **SMS printer costs per specimen to expand coverage** | **$0.82** | = ($63,240) / (76,752 specimens) | **= A** |

## Step 3 / Calculating per specimen training costs

| **Table D3** | | |  |  |  |
| --- | --- | --- | --- | --- | --- |
| **Training costs per person per day** | | |  |  |  |
| Meals | | $20 |  |  |  |
| Transport | | $20 |  |  |  |
| Accommodations | | $25 |  |  |  |
| Venue | | $20 |  |  |  |
| **SUM** | | $85 |  |  |  |
| **Calculating per specimen training costs** | | | | | |
| Training costs per person per day | $85 | | |  |  |
| People | 59 | | |  |  |
| Days | 4 | | |  |  |
| Total costs | $20,060 | | | = ($85)*(59 people)*(4 days) |  |
| **Per specimen training costs** | **$0.26** | | | **= ($20,060) / (76,752 annual specimens)** | **= B** |

Step 4 / Additional per sample transportation costs

| **Table D4** |  |  |  |  | | | |
| --- | --- | --- | --- | --- | --- | --- | --- |
| **Type of cost** | **Weekly transport** |  | **Daily transport** | **Detail** | | | |
| *True per specimen costs (multiplied 5 (5 weekdays) to reflect change in cost required for increasing from weekly to daily transport* | | | | | | | |
| Fuel | $ 0.48 | x 5 | $ 2.40 |  | | | |
| Investment costs | $ 0.27 | x 5 | $ 1.36 |  | | | |
| Insurance | $ 0.016 | x 5 | $ 0.08 |  | | | |
| Service & maintenance | $ 0.54 | x 5 | $ 2.69 |  | | | |
| *Costs that increased, but did not directly correlate 1:1 with specimens* | | | | | | | |
| Personnel | $ 1.17 |  | $ 5.72 | *Not all staff needed to be increased to facilitate weekly🡪daily shift; staffing for motor bike riders did increase, while central coordinator staff costs remained constant. | | | |
| Materials | $ 0.24 |  | $ 0.51 | *Not all materials needed to be increased to facilitate weekly🡪daily shift; since the number of specimens remains constant between the existing *LAB* scenario and the modeled *S-LAB* scenario, specimen-specific costs did not increase. However, material costs related to increased transport, such as motorbikes, did increase. | | | |
| *Costs not affected by weekly🡪daily shift* | | | | | | | |
| Sample & result tracking | $ 0.10 |  | **-** | *Existing sample tracking systems sufficient for increased load. No additional costs needed. | | | |
| **SUM** | **$ 2.82** | **SUM** | **$ 12.76** |  | | | |
| **Calculating additional per specimen transportation cost** | | | | | | | |
| Cost under daily transport system: | | | | | $ 12.76 |  |  |
| Cost under weekly transport system: | | | | | $ 2.82 |  |  |
| **Additional per specimen cost incurred by increasing from weekly to daily transport** | | | | | **$ 9.94** | **= ($12.76) - ($2.82)** | **= C** |

Step 5 / Calculating additional per specimen salary costs

| **Table D5** | | | |
| --- | --- | --- | --- |
| **Additional salary costs under strengthened system** | | | |
| *Lab scientists at central laboratory** |  |  | |
| Annual salary | $20,880 | Currently, 2 lab scientists per machine. Under a strengthened system, 1 additional scientist per machine would accelerate TAT | |
| # of EID machines in Zimbabwe | 5 | Currently, 5 machines across the 3 conventional labs | |
| Total additional annual salary for lab scientists | $104,400 | = ($20,880) * (5 machines) | |
| *Data officers at testing labs** |  |  | |
| Annual salary | $8,400 | An additional data officer per testing lab would accelerate dispatch | |
| # conventional labs in Zimbabwe | 3 | NMRL; Mutare; Mpilo | |
| Total additional annual salary for data officers | $25,200 | = ($8,400) * (3 testing labs) | |
| **Calculating additional per specimen costs** | | | |
| Total additional annual salary for lab scientists | $104,400 |  |  |
| Total additional annual salary for data officer | $25,200 |  |  |
| Total additional annual salary costs | $129,600 | = ($25,200) + ($104,400) |  |
| **Per specimen salary costs** | **$1.69** | **= ($129,600) / (76,752 samples)** | **= D** |

*Note: these are dedicated EID staff, with 100% of time going to EID activities

Step 6 / Sum calculated per specimen costs

| **Table D6** | | |
| --- | --- | --- |
| **Annual per specimen costs for strengthening existing EID system** | | |
| A | SMS printer costs to expand coverage | $0.82 |
| B | SMS printer maintenance training costs | $0.26 |
| C | Additional transport cost required to increase from daily to weekly transport | $9.94 |
| D | Additional salary costs | $1.69 |
| **Total costs for strengthening (per specimen)** | | **$12.71** |

Step 7 / Add total per specimen costs required for strengthening to the original per test costs within the pre-strengthened system

| **Table D7** | |
| --- | --- |
| **Cost** | **Description** |
| $17.76 | Per specimen cost under the original central laboratory-based scenario, including **weekly transportation** costs, excluding site monitoring |
| $12.71 | Per specimen cost of strengthening, including **additional** transportation costs associated with increasing sample transport from weekly to daily |
| **$30.47** | **Final per specimen cost under the strengthened central laboratory-based scenario** |

**Appendix Table E. Per test cost of POC EID**

| **Cost component** | **GeneXpert Gel** | **Abbott RDxmPima- reagent rental** |
| --- | --- | --- |
| Materials and Supplies | $19.12 | $21.49 |
| Training | $0.27 | $0.27 |
| Facility upgrades and repairs | $0.22 | $0.22 |
| Site monitoring and supervision | $0.34 | $0.34 |
| Equipment, shipping, and freight | $3.17 | N/A |
| Labor | $2.18 | $1.95 |
| Error rate | 7.8% | 6.7% |
| Sample transport | $3.44 | $3.44 |
| **Total cost per valid test** | $30.71 | $29.33 |

This table is adapted from Mukherjee et al. 2020, submitted to *JAIDS*

**Appendix Figure A. Comparison of EID strategies**


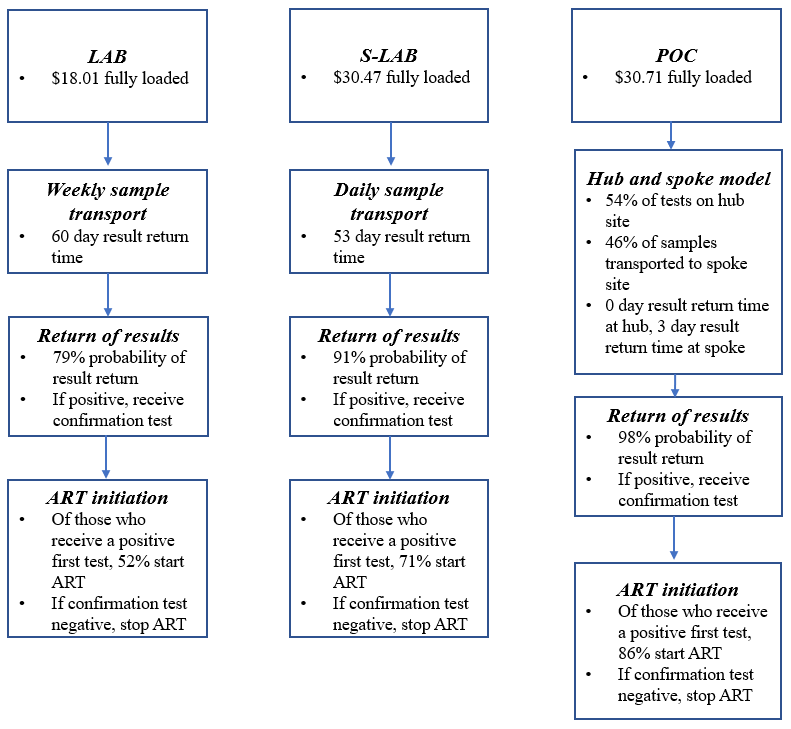


**LEGEND**

**Appendix Figure A. Comparison of EID strategies**

Stepwise comparison of *LAB, S-LAB,* and *POC. LAB/S-LAB/POC.* Algorithms differed in: total cost / test ($18.10/$30.47/$30.71), probability of result return (79%/91%/98%), time until result return (61/53/1 days), and probability of initiating ART after positive result (52%/71%/86%). Compared to *LAB, S-LAB* had more frequent sample transport, and *POC* used a hub and spoke structure.

**REFERENCES**

1. Francke JA, Penazzato M, Hou T, et al. Clinical impact and cost-effectiveness of diagnosing HIV infection during early infancy in South Africa: test timing and frequency. *J Infect Dis*. 2016;214(9):1319-1328. doi:10.1093/infdis/jiw379

2. Dunning L, Francke JA, Mallampati D, et al. The value of confirmatory testing in early infant HIV diagnosis programmes in South Africa: A cost-effectiveness analysis. *PLoS Med*. 2017;14(11):e1002446. doi:10.1371/journal.pmed.1002446

3. Frank SC, Cohn J, Dunning L, et al. Clinical effect and cost-effectiveness of incorporation of point-of-care assays into early infant HIV diagnosis programmes in Zimbabwe: a modelling study. *Lancet HIV*. 2019;6(3):e182-e190. doi:10.1016/S2352-3018(18)30328-X

4. Elizabeth Glaser Pediatric AIDS Foundation. UNITAID and Elizabeth Glaser Pediatric AIDS Foundation Launch Initiative to Significantly Scale Up HIV Diagnosis Among Infants in Africa. http://www.pedaids.org/press/entry/unitaid-and-elizabeth-glaser-pediatric-aids-foundation-launch-initiative-to. Accessed November 6, 2019.

5. Bailey R. Integrating New Point-of-Care Technologies into National Early Infant Diagnostic Networks. Elizabeth Glaser Pediatric AIDS Foundation. http://www.pedaids.org/2015/12/21/integrating-new-point-of-care-technologies-into-national-early-infant-diagnostic-networks/. Published 2015. Accessed November 1, 2019.

6. Bianchi F, Cohn J, Sacks E, et al. Evaluation of a routine point-of-care intervention for early infant diagnosis of HIV: an observational study in eight African countries. *Lancet HIV*. 2019;6(6):e373-e381. doi:10.1016/S2352-3018(19)30033-5

7. Elizabeth Glaser Pediatric AIDS Foundation. Catalyzing Expanded Access to Early Testing, Care and Treatment among HIV-Exposed Infants. http://www.pedaids.org/pages/unitaid-egpaf-project-optimizing-early-infant-diagnosis-and-treatment-for-h. Published 2015. Accessed October 31, 2019.

8. Ciaranello AL, Morris BL, Walensky RP, et al. Validation and calibration of a computer simulation model of pediatric HIV infection. *PLoS ONE*. 2013;8(12):e83389. doi:10.1371/journal.pone.0083389

9. Medical Practice Evaluation Center. Using the CEPAC Model to Simulate HIV progression and Outcomes. Massachusetts General Hospital. http://www.massgeneral.org/mpec/cepac/.

10. Ciaranello AL, Doherty K, Penazzato M, et al. Cost-effectiveness of first-line antiretroviral therapy for HIV-infected African children less than 3 years of age. *AIDS*. 2015;29(10):1247-1259. doi:10.1097/QAD.0000000000000672

11. Ciaranello AL, Lockman S, Freedberg KA, et al. First-line antiretroviral therapy after single-dose nevirapine exposure in South Africa: a cost-effectiveness analysis of the OCTANE trial. *AIDS*. 2011;25(4):479-492. doi:10.1097/QAD.0b013e3283428cbe

12. Walensky RP, Ross EL, Kumarasamy N, et al. Cost-effectiveness of HIV treatment as prevention in serodiscordant couples. *N Engl J Med*. 2013;369(18):1715-1725. doi:10.1056/NEJMsa1214720

13. Dabis F, Bequet L, Ekouevi DK, et al. Field efficacy of zidovudine, lamivudine and single-dose nevirapine to prevent peripartum HIV transmission. *AIDS*. 2005;19(3):309-318.

14. Marston M, Becquet R, Zaba B, et al. Net survival of perinatally and postnatally HIV-infected children: a pooled analysis of individual data from sub-Saharan Africa. *Int J Epidemiol*. 2011;40(2):385-396. doi:10.1093/ije/dyq255

15. Ciaranello A, Lu Z, Ayaya S, et al. Incidence of World Health Organization stage 3 and 4 events, tuberculosis and mortality in untreated, HIV-infected children enrolling in care before 1 year of age: an IeDEA (International Epidemiologic Databases To Evaluate AIDS) East Africa regional analysis. *Pediatr Infect Dis J*. 2014;33(6):623-629. doi:10.1097/INF.0000000000000223

16. Violari A, Lindsey JC, Hughes MD, et al. Nevirapine versus ritonavir-boosted lopinavir for HIV-infected children. *N Engl J Med*. 2012;366(25):2380-2389. doi:10.1056/NEJMoa1113249

17. Palumbo P, Lindsey JC, Hughes MD, et al. Antiretroviral treatment for children with peripartum nevirapine exposure. *N Engl J Med*. 2010;363(16):1510-1520. doi:10.1056/NEJMoa1000931

18. Losina E, Yazdanpanah Y, Deuffic-Burban S, et al. The independent effect of highly active antiretroviral therapy on severe opportunistic disease incidence and mortality in HIV-infected adults in Côte d’Ivoire. *Antivir Ther (Lond)*. 2007;12(4):543-551.

19. Nichols BE, Girdwood SJ, Crompton T, et al. Monitoring viral load for the last mile: what will it cost? *J Int AIDS Soc*. 2019;22(9):e25337. doi:10.1002/jia2.25337

20. Iliff PJ, Piwoz EG, Tavengwa NV, et al. Early exclusive breastfeeding reduces the risk of postnatal HIV-1 transmission and increases HIV-free survival. *AIDS*. 2005;19(7):699-708. doi:10.1097/01.aids.0000166093.16446.c9

21. Dinh T-H, Mushavi A, Shiraishi RW, et al. Impact of timing of antiretroviral treatment and birth weight on mother-to-child human immunodeficiency virus transmission: findings from an 18-month prospective cohort of a nationally representative sample of mother-infant pairs during the transition from Option A to Option B+ in Zimbabwe. *Clin Infect Dis*. 2018;66(4):576-585. doi:10.1093/cid/cix820

22. Lilian RR, Johnson LF, Moolla H, Sherman GG. A mathematical model evaluating the timing of early diagnostic testing in HIV-exposed infants in South Africa. *J Acquir Immune Defic Syndr*. 2014;67(3):341-348. doi:10.1097/QAI.0000000000000307

23. Zimbabwe Demographic and Health Survey 2015. https://www.dhsprogram.com/pubs/pdf/SR234/SR234.pdf. Accessed October 14, 2019.

24. Zimbabwe Population-Based HIV Impact Assessment 2015-2016. ZIMPHIA. https://phia.icap.columbia.edu/wp-content/uploads/2016/11/ZIMBABWE-Factsheet.FIN_.pdf. Accessed November 4, 2019.

25. Stover J, Glaubius R, Mofenson LM, et al. *Updates to the Spectrum/AIM Model for Estimating Key HIV Indicators at National and Sub-National Levels*. AIDS, in press; 2019.

26. EID consortium. Field Performance of Point-of-Care HIV Testing for Early Infant Diagnosis: Pooled analysis from six countries from the EID consortium. https://eidconsortium.org/Files/EID%20Poster%20v5%20Low%20res.pdf. Accessed February 27, 2020.

27. Mukherjee S, Cohn J, Ciaranello AL, et al. Estimating the cost of point-of-care early infant diagnosis in a program setting: a case study using Abbott RDx m-PIMA and Cepheid GeneXpert in Zimbabwe. *Submitted to Journal of Acquired Immune Deficiency Syndromes*. 2019.

28. Holmes CB, Wood R, Badri M, et al. CD4 decline and incidence of opportunistic infections in Cape Town, South Africa: implications for prophylaxis and treatment. *J Acquir Immune Defic Syndr*. 2006;42(4). doi:10.1097/01.qai.0000225729.79610.b7

29. Becquet R, Marston M, Dabis F, et al. Children who acquire HIV infection perinatally are at higher risk of early death than those acquiring infection through breastmilk: a meta-analysis. *PLoS ONE*. 2012;7(2):e28510. doi:10.1371/journal.pone.0028510

30. United Nations. World Population Prospects: The 2008 Revision New York 2009. http://www.un.org/esa/population/publications/wpp2008/wpp2008_highlights.pdf. Accessed December 12, 2019.

31. Mallampati D, Ford N, Hannaford A, Sugandhi N, Penazzato M. Performance of virological testing for early infant diagnosis: a systematic review. *J Acquir Immune Defic Syndr*. 2017;75(3):308-314. doi:10.1097/QAI.0000000000001387

32. Hsiao N, Dunning L, Kroon M, Myer L. Laboratory evaluation of the Alere q point-of-care system for early infant HIV diagnosis. *PLoS ONE*. 2016;11(3):e0152672. doi:10.1371/journal.pone.0152672

33. Walmsley SL, Antela A, Clumeck N, et al. Dolutegravir plus abacavir-lamivudine for the treatment of HIV-1 infection. *N Engl J Med*. 2013;369(19):1807-1818. doi:10.1056/NEJMoa1215541

34. Barth RE, van der Loeff MFS, Schuurman R, Hoepelman AIM, Wensing AMJ. Virological follow-up of adult patients in antiretroviral treatment programmes in sub-Saharan Africa: a systematic review. *Lancet Infect Dis*. 2010;10(3):155-166. doi:10.1016/S1473-3099(09)70328-7

35. Ciaranello AL, Chang Y, Margulis AV, et al. Effectiveness of pediatric antiretroviral therapy in resource-limited settings: a systematic review and meta-analysis. *Clin Infect Dis*. 2009;49(12):1915-1927. doi:10.1086/648079

36. Mabugu T. *Zimbabwe National AIDS Spending Assessment: Consolidated Report 2011 and 2012*. UNAIDS

37. Menzies NA, Berruti AA, Berzon R, et al. The cost of providing comprehensive HIV treatment in PEPFAR-supported programs. *AIDS*. 2011;25(14):1753-1760. doi:10.1097/QAD.0b013e3283463eec

38. Clinton Health Access Initiative. *HIV/AIDS Diagnostic Pricing Outlook.*; 2009.

39. HIV Viral Load and Early Infant Diagnosis Selection and Procurement Information Tool. The Global Fund to Fight AIDS, Tuberculosis and Malaria. https://www.theglobalfund.org/media/5765/psm_viralloadearlyinfantdiagnosis_content_en.pdf. Published April 2017. Accessed October 1, 2019.

40. Clinton Health Access Initiative. *2017 CHAI ARV Reference Price List*.; 2017.
